# Tics in the Pediatric Population: Pragmatic Management

**DOI:** 10.1002/mdc3.12428

**Published:** 2016-11-11

**Authors:** Christos Ganos, Davide Martino, Tamara Pringsheim

**Affiliations:** ^1^ Department of Neurology University Medical Center Hamburg‐Eppendorf Hamburg Germany; ^2^ Sobell Department of Motor Neuroscience and Movement Disorders University College London Institute of Neurology University College London London United Kingdom; ^3^ International Parkinson's Centre of Excellence King's College and King's College Hospital Denmark Hill Campus London United Kingdom; ^4^ Queen Elizabeth Hospital, Woolwich Lewisham and Greenwich National Health Service Trust London United Kingdom; ^5^ Department of Clinical Neurosciences University of Calgary Calgary Alberta Canada

**Keywords:** Tourette syndrome, primary tic disorder, habit reversal training, pharmacological treatment, antipsychotics

## Abstract

**Background:**

Primary tic disorders, notably Tourette syndrome, are very common movement disorders in childhood. However, the management of such patients still poses great therapeutic challenges to medical professionals.

**Methods:**

Based on a synthesis of the available guidelines published in Europe, Canada, and the United States, coupled with more recent therapeutic developments, the authors provide a pragmatic guide to aid clinicians in deciding when and how to treat patients who have primary tic disorders.

**Results:**

After a systematic assessment of tics and common neuropsychiatric comorbidities (primarily attention‐deficit hyperactivity disorder [ADHD] and obsessive‐compulsive disorder [OCD]), the first step in treatment is a comprehensive psychoeducation of patients and families that addresses the protean phenomenology of tics and associated behaviors, coping mechanisms, prognosis, and treatment options. When more active intervention beyond watchful monitoring is indicated, hierarchical evaluation of treatment targets (i.e., tics vs. comorbid behavioral symptoms) is crucial. Behavioral treatments for tics are restricted to older children and are not readily available to all centers, mainly due to the paucity of well‐trained therapists. Pharmacological treatments, such as antipsychotics for tics, stimulants and atomoxetine for ADHD, and α2A‐agonists for children with tics plus ADHD, represent widely available and effective treatment options, but safety monitoring must be provided. Combined polypharmacological and behavioral/pharmacological approaches, as well as neuromodulation strategies, remain under‐investigated in this population of patients.

**Conclusions:**

The treatment of children with tics and Tourette syndrome is multifaceted. Multidisciplinary teams with expertise in neurology, psychiatry, psychology, and pediatrics may be helpful to address the complex needs of these children.

Tics are brief and sudden movements or sounds that may be indistinguishable from physiological actions but appear repetitive, often disruptive, and are not embedded in a certain context but can be inhibited on demand.^1^ Tics are classified as a hyperkinetic movement disorder; however, different from other hyperkinesias, they are typically preceded by a premonitory sensation known as the “premonitory urge.” Tics may present in a wide range of different neuropsychiatric conditions across the lifespan but are most often encountered in children and adolescents with primary tic disorders, and particularly in Tourette syndrome (TS). According to the *Diagnostic and Statistical Manual for Disease*, 5th edition (DSM‐5), TS is defined by the presence of both tic movements and sounds that appear before age 18 years and last for more than 1 year, in the absence of substance abuse or another medical condition.^2^

Tics are not a rare phenomenon in the general population, particularly in children. Prevalence estimates for all tic disorders, including transient tic disorders (i.e., tics appearing before age 18 years and being present for less than 1 year), in pediatric populations approach 3%^3^; and, for TS in particular, they approach 1%.^3^, ^4^ In adults, tics appear to be less common.^3^ However, the scarcity of systematic, large‐scale studies within this age group suggests that existing prevalence rates may reflect underestimates. Nevertheless, the natural course of primary tic disorders supports the increased prevalence rates in childhood, because the severity, complexity, and frequency of tics do attenuate in adulthood, at least for some patients.^5^, ^6^ Poor clinical predictors for future tic outcomes appear to be tic severity during childhood and the presence of common neuropsychiatric comorbidities, such as attention‐deficit hyperactivity disorder (ADHD) or obsessive‐compulsive disorder (OCD).^5^, ^6^

Indeed, the majority of patients with TS will have signs of comorbid neuropsychiatric conditions (hereinafter termed “TS plus”). Although prevalence estimates vary largely and depend on the selection criteria of the studied population and ascertainment methods, ADHD and OCD reportedly affect up to two‐thirds of patients with TS.^7^ Other common conditions include anxiety disorders, depression and other mood disorders, autism spectrum disorder, conduct disorder, oppositional defiant disorder, self‐injurious behavior, sleep disorder, and personality disorder.^1^, ^7^, ^8^ In fact, only about 15% of patients with TS have isolated motor and/or phonic tics, also termed “pure” or uncomplicated TS.^7^ It is noteworthy that, according to those authors' experience, a significant proportion of these patients will still exhibit subclinical signs of some of the aforementioned conditions.

Despite the common nature of tics, the treatment of patients with TS often falls short between the disciplinary trenches of (pediatric) neurology, psychiatry, and psychology. This may be due to the unusual characteristics of tics, with their protean phenomenology and variable severity, as well as the challenging neuropsychiatric profiles of patients with TS. To overcome these difficulties, treatment guidelines have been published in Europe,^9^ Canada,^10^ and the United States.^11^ These guidelines were based on systematic literature reviews as well as expert consensus, and they provide levels of evidence and practice recommendations for behavioral, pharmacological, and surgical treatments. However, the usually young age of patients, the great variation in clinical presentation, and the range of the different behavioral problems that such patients may face still pose great therapeutic challenges to medical professionals. Based on a synthesis of the available recommendations and more recent therapeutic developments,^12^, ^13^ as well as our own experience, we focus this review on the treatment of tics and associated neuropsychiatric disorders in the most commonly affected, pediatric population. Our goal is to provide a pragmatic guide that will aid clinicians in deciding when to treat patients with primary tic disorders, which symptoms to address first, and which treatment options to consider.

## Prioritizing Treatment Goals in the Child with TS

Clinicians who treat children with TS must first evaluate the presence of neuropsychiatric comorbidities, such as ADHD, OCD, autism spectrum disorder, depression, anxiety disorders, oppositional defiant disorder, rage attacks, etc., and subsequently must assess symptom severity and symptom‐related disability in each separate symptom domain. This evaluation can be facilitated through the use of standardized measures of symptom severity, including the Yale Global Tic Severity Scale (YGTSS) for tics,^14^ Conner's 3‐Parent Form for ADHD,^15^ and the Children's Yale‐Brown Obsessive Compulsive Scale for OCD.^16^ The child and parent also should be asked directly about their treatment priorities and which symptoms they find most disabling; and, indeed, the treatment of children with tics and TS should be individualized. However, studies suggest that treatment for symptoms of ADHD and OCD should be the priority in children who have “TS plus,” because, in such children, these symptoms account for most of the impairment in psychosocial health.^17^, ^18^ Once the goals of consultation and treatment are established with the child and family, it is advisable to address treatment targets sequentially, recognizing that some treatments may provide benefits in multiple symptom domains.

## The Treatment of Tics

### Psychoeducation

**Figure 1:**
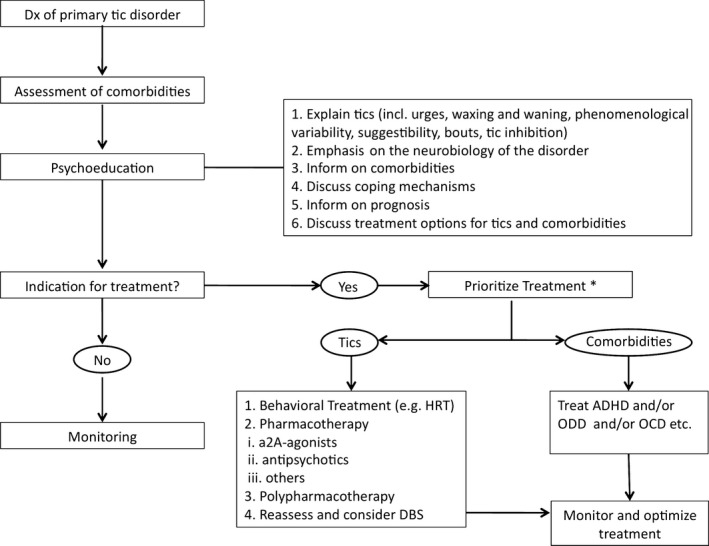
This is a flowchart of treatment options for patients with Tourette syndrome. An asterisk indicates that behavioral comorbidities should be treated first if they are a major source of disability; then, proceed to the treatment of tics at follow‐up. Dx, diagnosis; HRT, habit reversal training; ODD, oppositional defiant disorder; DBS, deep‐brain stimulation.

In patients with primary tic disorders, the cornerstone of treatment is psychoeducation. This first step is often the only necessary treatment intervention and is essential to inform patients and their families about the neurobiological basis of the condition; its natural history, including the waxing and waning of tics; relevant contextual factors (e.g., suggestibility, the role of stress and fatigue, etc.); as well as of the spectrum of associated neuropsychiatric symptoms. Indeed, families often find it difficult to reconcile their immediate experiences, because they witness the temporary voluntary control that their child can exert on tics with the model of a neurobiological disorder that places abnormal tics beyond the child's control. In addition, guilt and blame may also surface, because parents often may feel responsible for the disruptive motor behaviors of their children. Therefore, psychoeducation is crucial to facilitate understanding the nature of the disorder, unravel relevant family dynamics, and ultimately increase symptom awareness and acceptance for patients and their families. Psychoeducation can thereby establish an open discourse on the relevant difficulties that patients and families may face, and potential coping strategies can be discussed. Indeed, the importance of psychoeducation is recognized in all 3 of the aforementioned treatment guidelines from Europe, Canada, and the United States^9^, ^10^, ^11^ (see also Fig. 1).

However, in some cases, behavioral and/or pharmacological treatments for tics also will be required. For example, these are patients whose tics are painful or injurious, or might directly cause functional impairment, and/or constitute a source of social distress, leading, in turn, to impairment of quality of life and mood and/or anxiety disorders.^9^ At this point, whether nonpharmacological treatments should be preferred as a first‐line option over pharmacotherapy is a debated issue, and the decision often depends not only on clinical aspects (e.g., tic severity) but also practical aspects, including availability and cost coverage of the former over the latter. Given the potential drawbacks of medication therapies, which are mostly associated with drug‐induced side effects in pediatric patients, below, we describe behavioral therapies first and then pharmacological interventions.

### Behavioral Therapies

The nonpharmacological treatment of tics has involved numerous therapies over the course of time, including psychoanalytic psychotherapy, relaxation training, and hypnosis, but also massed negative practice, self‐monitoring, habit‐reversal training (HRT), exposure‐response prevention (ERP), contingency management, biofeedback, mindfulness‐based approaches, acceptance and commitment therapy, and others.^19^ Among these approaches, both HRT and ERP have received strong or Class A recommendations for the treatment of tics by the Canadian and European guidelines.^10^, ^19^ HRT is also prioritized over pharmacological treatments in the published US practice parameter.^11^

HRT consists of different elements, with an emphasis on awareness the of somatic/interoceptive^20^ signals (premonitory urges) that precede disruptive tics and competing response training, which focuses on the early implementation of behaviors that are physically incompatible with these tics.^21^ This, in turn, leads to an interruption of a postulated cycle of negative reinforcement between an urge and a tic, leading to their habituation. HRT is also the main therapeutic element of the Comprehensive Behavioral Intervention for Tics (CBIT), which also involves relaxation training as well as a “functional intervention,” which addresses situations that influence tic severity. To date, only 1 randomized control trial (RCT) has assessed the efficacy of CBIT compared with supportive psychotherapy alone in a relatively large number of children and adolescents (n = 126; ages 9–17 years: n = 65 who received 8 CBIT sessions over 10 weeks [baseline tic severity, 24.7 points on the YGTSS] vs n = 61 who received supportive psychotherapy [baseline tic severity, 21.1 points]; follow‐up to 6 months post‐treatment).^22^ It is noteworthy that 36.5% of these patients were receiving medication for tics at the time of the study, and a significant proportion of participants had TS‐associated comorbidities. At Week 10, patients who received CBIT showed a 30.8% improvement in tic severity compared with 14.2% in patients who received supportive therapy (*P* < 0.001). Importantly, 86.9% of patients who received CBIT showed continued benefit at the 6‐month follow‐up assessment. Based on those results and also on evidence from smaller studies in children and adults (reviewed by McGuire et al.^23^), HRT should be considered as first‐line intervention for tics in children and adolescents with TS, particularly in cases where discrete, isolated tics are the cause of pain or major functional/social impairment.

Similar to HRT, ERP proposes that tics are conditioned responses to unpleasant premonitory urges, which are strengthened by repetition, as a result of associative learning.^19^ ERP aims to interrupt this association by training patients to tolerate premonitory urges until they habituate to them while suppressing tics for prolonged time periods. A single RCT by Verdellen et al. compared ERP versus HRT in 43 children and adults (ages 7–55 years) and found significant improvements in total tic severity on the YGTSS between baseline and the endpoint in both treatment groups, with no significant differences between the treatments.^24^

Significant limitations for both HRT and ERP constitute the age of patients, tic severity, comorbidity profile (especially severe ADHD),^23^ treatment availability, and cost coverage. Indeed, particularly young children (those younger than <10 years) may not yet be aware of premonitory urges and may not understand the content of treatments, hence limiting the efficacy of such interventions. In addition, severely affected patients may not be able either to focus on a particular urge associated with a certain tic (HRT) or to suppress their tics for more than very few seconds, if at all (ERP). Finally, despite the rising potential of telehealth approaches,^25^ to date, nonpharmacological interventions strongly depend on the abilities and experience of individual therapists; therefore, they may not be available in different parts of the world. In addition, therapy costs may not be covered by standard health care, further complicating access to these treatment programs.

### Pharmacotherapy

Two different classes of medication are currently used as first‐line in the pharmacotherapy of multifocal tics in childhood: α2‐adrenergic agonists and antipsychotics. Other relevant medications for patients of this age group include tetrabenazine, topiramate and potentially baclofen. Although botulinum toxin may provide a very useful treatment approach for focal tics in older adolescents and adults,^26^ its use in children is limited due to a general aversion in this population to injections. We recommend it only in cases with particularly harmful (malignant) focal tics, such as whiplash tics or disabling blinking, and in the hands of experienced movement disorders specialists.

Clonidine and guanfacine are α2‐adrenergic agonists used in the treatment of (childhood) tics. A survey on first‐line treatment strategies of tics in TS among European experts identified clonidine as the second most commonly prescribed agent.^9^ Similarly, in a single‐clinic sample of 255 patients with TS (77 children) who received medication for tics, clonidine reportedly was second in prescription frequency after aripiprazole.^27^ Indeed, upon reviewing the available evidence and considering the milder and reversible spectrum of associated side effects (see below), the Canadian guidelines and the US practice parameter for the treatment of childhood tics made strong recommendations for both clonidine and guanfacine as first‐line treatment over antipsychotics.^10^, ^11^ It is worth noting that a randomized, placebo lead‐in, double‐blind study comparing risperidone with clonidine in the treatment of tics in 21 children and adolescents with TS showed comparable efficacy for both substances over an 8‐week period.^28^ Similarly, clonidine (n = 128) and haloperidol (n = 116) were found to be equally effective in reducing tics in a recent 4‐week RCT in children ages 5 to 12 years with TS.^29^ However, a careful meta‐analysis of the existing studies showed that clonidine might be effective only in patients with comorbid ADHD^30^; and, indeed, the European guidelines favored antipsychotics over α2‐adrenergic agonists as first‐line tic‐suppressing agents.^9^ These findings indicate that clinicians who are considering the use of α agonists for tic suppression should evaluate patients for the presence of comorbid ADHD and should consider treatment options carefully for patients without ADHD. Given the more favorable risk profile of α agonists relative to antipsychotics, the strong recommendations for their use as first‐line therapy in the Canadian and US guidelines is logical. Clinicians may explain this potential limitation in the evidence to patients and families who are considering treatment so that an informed choice can be made regarding initial medication. Patients who are receiving clonidine and guanfacine should be monitored for side effects, such as reduced blood pressure and orthostatic hypotension, bradycardia, sedation, dizziness, irritability, and interrupted night sleep; moreover, both substances should be titrated gradually to minimize potential side effects and should be discontinued gradually to avoid rebound hypertension.

Antipsychotics have been systematically used in the treatment of tics for over 40 years.^31^, ^32^ Based on current evidence, they are the best‐studied and most efficacious substances for reducing the severity and frequency of tics.^9^, ^10^, ^11^ The following antipsychotics have been evaluated for the treatment of tics: haloperidol, pimozide, fluphenazine, risperidone, aripiprazole, tiapride, sulpiride, olanzapine, ziprasidone, quetiapine, and ecopipam.^9^, ^10^, ^33^ Among those, haloperidol and pimozide, which are first‐generation (or typical) antipsychotics, have been studied in comparatively large numbers of patients, including children and adolescents, leading to meaningful tic improvement. Fluphenazine, another typical antipsychotic, reportedly led to moderate tic improvement in about 80% of patients with overall relatively good tolerability in a retrospective chart review of 268 pediatric and adult patients over a period of 26 years.^34^ However, the presence of potentially serious adverse effects associated with typical antipsychotics, such as drug‐induced movement disorders (e.g., akathisia, parkinsonism, acute dystonia), drowsiness and sedation, metabolic effects, as well as prolongation of the QTc interval for pimozide, limits their use for tic disorders, particularly in children.

Within the group of second‐generation antipsychotics, risperidone and aripiprazole are first‐line treatment choices based on European expert recommendations^9^ and are indeed most commonly used.^27^ Efficacy for both of these substances has been well demonstrated in several studies, including randomized placebo controlled trials.^28^, ^35^, ^36^, ^37^, ^38^, ^39^, ^40^, ^41^, ^42^, ^43^, ^44^, ^45^ Typical side effects for risperidone in the context of tic disorders include sedation/somnolence, weight gain and associated metabolic disturbances, hyperprolactinemia and associated gynecomastia, galactorrhea and menstrual cycle disturbances, and, less frequently, drug‐induced movement disorders, such as acute dystonic reactions and parkinsonism. Therefore, monitoring for neuropsychiatric side effects as well as movement, metabolic, and hormonal disorders should be guaranteed. For aripiprazole, reported adverse effects include nausea, sedation, somnolence, moderate weight gain and metabolic disturbances, as well as movement disorders. In this context, benzamides, such as tiapride and sulpiride, comprise another class of substances to highlight in this context. Although neither substance is available in Canada and the United States, they are often favored in Europe due to their demonstrated efficacy^46^, ^47^, ^48^, ^49^ and their relatively mild side‐effect profile (sedation, increased appetite with weight gain, as well as transient hyperprolactinemia). Indeed, tiapride is recommended as first‐line treatment in the existing German guidelines for the pharmacotherapy of tics.^50^ It is noteworthy that ziprasidone also significantly reduced tic severity in a small sample of patients ages 7 to 17 years at an average dosage of 28.2 mg without leading to weight gain. Transient somnolence was the most common side effect associated with ziprasidone.^51^ Monitoring the QTc interval in patients who are receiving ziprasidone is recommended.

Despite the apparent abundance of different antipsychotic agents for the treatment of tics, 2 recent meta‐analyses did not detect meaningful differences between these compounds in terms of tic reduction in children and adolescents.^30^, ^52^ In children, the bulk of experience with antipsychotic medications is with risperidone and aripiprazole, which have been studied in RCTs not only for tic disorders but also in youths with autism, ADHD, and ODD. Given the greater amount of data on the safety and efficacy of risperidone and aripiprazole in children and youths, their use is favored over other antipsychotics, recognizing that an individualized approach to the selection of the appropriate medication is necessary for these young patients. The approach should consider not only the treatment of tics but also the profile of relevant comorbidities (see also below) as well as the spectrum of potential drug‐induced side effects.

Tetrabenazine blocks vesicular monoamine transporter type 2 (VMAT2), leading to the depletion of presynaptic dopamine with relatively minor postsynaptic dopamine receptor blockage. Although data on its efficacy over tics are limited to 2 open‐label trials and retrospective cohort studies, it has been demonstrated that tetrabenazine significantly reduces the severity of tics in >75% of pediatric and adult patients.^53^ Adverse effects include drowsiness/fatigue, nausea, depression, akathisia and parkinsonism, as well as insomnia.^53^, ^54^, ^55^, ^56^ Due to an acceptable tolerability profile and its efficacy supported by noncontrolled studies, some authorities in the field include tetrabenazine among first‐line agents.^54^, ^55^, ^56^ One RCT assessed the efficacy of topiramate for the treatment of tics in a small sample of 29 patients ages 7 to 65 years.^57^ Twenty participants completed that study, and there was a significant decrease in the YGTSS total tic score of 14.3 with topiramate (average daily dosage, 118 mg) versus 5.0 points with placebo (*P* = 0.03). Importantly, there was no difference in adverse effects.^57^ According to the Canadian guidelines, topiramate received a weak recommendation based on low‐quality evidence.^10^ Finally, a large, open‐label study found a significant reduction in YGTSS‐rated motor and phonic tics in 250 of 264 children and adolescents (ages 6–18 years) with primary tic disorders, including TS, who received baclofen over a period of 4 weeks.^58^ However, a more recent small, double‐blind, randomized, placebo‐controlled study in 10 children ages 8 to 14 years over 4 weeks did not provide conclusive results.^59^ Baclofen‐related side effects included sedation, drowsiness, constipation, nausea, anxiety, and headache.^58^, ^59^ Tables 1 and 2 provide a list of dosages, side‐effect profiles, and monitoring recommendations for antipsychotic (Table 1) and non‐antipsychotic (Table 2) pharmacologic treatments for tics.^60^

**Table 1:** Selected evidence‐based antipsychotic treatments for tics in children and adolescents^a^

Medication	Daily Dose, mg	Adverse Effects	Recommended Monitoring	Level of Efficacy
First‐generation antipsychotics				
Haloperidol	0.5–3	Rigidity, parkinsonism, tardive involuntary movements and akathisia, appetite changes, weight gain, salivary changes, constipation, depression, anxiety, fatigue, sedation, hyperprolactinemia (galactorrhea, gynecomastia, irregular menses, sexual dysfunction)	*Physical examination*: Height, weight, BMI, waist circumference, blood pressure, assessment of drug‐induced movement disorders, ECG at baseline and at 3, 6, and 12 mo; *Laboratory tests*: Cell blood count, transaminase activity, prolactin	CG: High‐quality evidence, weak recommendation; ESSTS: Level A evidence
Pimozide	0.5–4	Similar to haloperidol but with less movement disorders; QTc interval prolongation	Same as for haloperidol	CG: High‐quality evidence, weak recommendation; ESSTS: Level A evidence
Fluphenazine	0.25–3	Similar to haloperidol but less frequent	Same as for haloperidol	CG: Low‐quality evidence, weak recommendation; ESSTS: Not provided
Atypical antipsychotics				
Aripiprazole	2–15	Moderate weight gain, increase in BMI and waist circumference, metabolic adverse effects, nausea, fatigue, sedation, movement disorders, sleep problems	*Physical examination*: Height, weight, BMI, waist circumference, blood pressure, assessment of drug‐induced movement disorders; *Laboratory tests*: Fasting plasma glucose, total cholesterol, LDL, HDL, fasting triglycerides, AST, and ALT at baseline and at 6 and 12 mo	CG: Moderate‐quality evidence, weak recommendation; ESSTS: Level C evidence
Risperidone	0.25–3	Sedation, fatigue, depression, weight gain, metabolic adverse effects, extrapyramidal side effects, hyperprolactinemia (gynecomastia, galactorrhea, menstrual irregularity)	*Physical examination*: Same as for aripiprazole, ECG at baseline and at 3, 6, and 12 mo; *Laboratory tests*: Fasting plasma glucose, insulin, total cholesterol, LDL, HDL, fasting triglycerides; prolactin at baseline and at 3, 6, and 12 mo; AST and ALT at baseline and at 6 and 12 mo	CG: High‐quality evidence, weak recommendation; ESSTS: Level A evidence
Tiapride/sulpiride	50–200	Sedation; less commonly, paradoxical depression, restlessness, sleep problems, weight gain, hyperprolactinemia	*Physical examination*: Same as for aripiprazole, ECG at baseline and at 3, 6, and 12 mo; *Laboratory tests*: Fasting plasma glucose, insulin, total cholesterol, LDL, HDL, fasting triglycerides; prolactin at baseline and at 3, 6, and 12 mo; AST and ALT at baseline and at 6 and 12 mo	CG: Not provided; ESSTS: Level B evidence
Ziprasidone	20–40	Sedation, anxiety, akathisia, movement disorders	*Physical examination*: Same as for aripiprazole, ECG at baseline and at 3, 6, and 12 mo; *Laboratory tests*: Fasting plasma glucose, total cholesterol, LDL, HDL, fasting triglycerides; ALT, AST, and prolactin at baseline and at 6 and 12 mo	CG: Low‐quality evidence, weak recommendation; ESSTS: Level B evidence
Olanzapine	2.5–10	Sedation, weight gain and increased appetite, metabolic adverse effects, dry mouth, transient hypoglycemia, extrapyramidal side effects	*Physical examination*: Same as for aripiprazole, ECG at baseline and at 3, 6, and 12 mo; *Laboratory tests*: Fasting plasma glucose, insulin, total cholesterol, LDL, HDL, fasting triglycerides; ALT, AST, and prolactin at baseline and at 3, 6, and 12 mo	CG: Low‐quality evidence, weak recommendation; ESSTS: Level B evidence
Quetiapine	50–250	Sedation, weight gain, metabolic adverse effects, akathisia, tremor	*Physical examination*: Same as for aripiprazole, ECG at baseline and at 3, 6, and 12 mo; *Laboratory tests*: Fasting plasma glucose, total cholesterol, LDL, HDL, fasting triglycerides; AST and ALT at baseline and at 3, 6, and 12 mo; prolactin and TSH at baseline and at 3 mo	CG: Very‐low‐quality evidence, weak recommendation; ESSTS: Level C evidence

a Modified from Roessner et al.,^9^ Pringsheim et al.,^10^ and Ganos and Martino.^60^

BMI, body mass index; ECG, electrocardiogram; CG, Canadian Guidelines; ESSTS; European Society for the Study of Tourette Syndrome; LDL, low‐density lipoprotein cholesterol; HDL, high‐density lipoprotein cholesterol; ALT, alanine aminotransferase; AST, aspartate aminotransferase; TSH, thyroid‐stimulating hormone.

**Table 2:** Nonantipsychotic treatments for tics in children and adolescents^a^

Medication	Daily Dose, mg	Adverse Effects	Recommended Monitoring	Level of Efficacy
Clonidine	Dosing should be titrated according to blood pressure and heart rate: 0.025–0.3	Sedation, bradycardia, orthostatic hypotension, dry mouth, headache, irritability, sleep disorder, rebound hypertension, tics and anxiety following abrupt discontinuation	Blood pressure, heart rate	CG: Moderate‐quality evidence, strong recommendation; ESSTS: Level A evidence^b^
Guanfacine	Dosing should be titrated according to blood pressure and heart rate: 0.5–3	Orthostatic hypotension, bradycardia, sedation, headache, fatigue, irritability, light headedness, stomach ache, and sleep disturbance	Blood pressure, heart rate	CG: Moderate‐quality evidence, strong recommendation; ESSTS: Level A evidence^b^
Tetrabenazine	12.5–50	Sedation, fatigue, nausea, insomnia, akathisia, parkinsonism, depression	Ensure normal hepatic function, monitor for depression	CG: Very‐low‐quality evidence, weak recommendation; ESSTS: Not provided
Topiramate	From 1 to 9 mg/kg/d; doses >200 mg are poorly tolerated	Weight loss, paresthesias	Monitor for cognitive side effects, mood changes, and weight loss	CG: Low‐quality evidence, weak recommendation; ESSTS: Not provided
Baclofen	10–60 mg^c^	Sedation, drowsiness, constipation, nausea, anxiety, and headache	—	CG: Weak recommendation, very‐low‐quality evidence
Botulinum toxin	Individualized therapy	Focal weakness, hypophonia	—	CG: Low‐quality evidence, weak recommendation
d‐9‐THC	Not recommended for use in children and adolescents			

a Modified from Roessner et al.,^9^ Pringsheim et al.,^10^ and Ganos and Martino.^60^

b Efficacy on tics is well documented only for patients with comorbid attention‐deficit hyperactivity disorder.

c Baclofen doses are up to 40 mg for patients aged 8 years and younger and up to 60 mg for those older than 8 years.

CG, Canadian Guidelines; ESSTS, European Society for the Study of Tourette Syndrome; d‐9‐THC, Δ^9^‐tetrahydrocannabinol.

### Tics Refractory to Behavioral and Pharmacological Treatments

Treatment refractoriness of tics to behavioral and pharmacological treatments is not yet a universally defined term in the TS literature.^61^ Even despite best treatment attempts with the aforementioned strategies, a proportion of patients will still suffer from clinically significant tics. Recently, Farag et al. assessed the numbers of medications attempted to treat tics in 255 patients who were seen at a single clinic.^27^ Interestingly, up to one‐third of those patients had tried 3 different anti‐tic compounds to achieve satisfactory tic control, and 21 had switched medications ≥5 times.^27^ In the absence of any good evidence on the best serial prescribing strategies for patients with TS, and even more so for polypharmacotherapy, these practices are still made based on individual experience, and only a consensus procedure or new evidence could integrate them in an algorithm.

Should all of the aforementioned treatment options not suffice, then deep‐brain stimulation (DBS) can be considered. According to the first published DBS recommendations for tics in patients with TS in 2006, the minimum patient age had to be 25 years.^62^ This was meant to ensure that symptoms would be stabilized by that age; hence, the risk of performing an invasive treatment like DBS on patients who would have improved simply by virtue of their age (and brain development), would be avoided. However, recognizing that, in some patients, tic burden, including potentially harmful tics, might hinder normal development, this age limit has been removed from the revised recommendation version.^63^ Although a review on the assessment and the potential targets of DBS in TS is beyond the scope of this pragmatic guide, it is worth noting that the revised DBS recommendation suggests trying at least 3 different categories of drugs, 2 of which should be α2A‐agonists and antipsychotics (suggesting 1 first‐line and 1 atypical antipsychotic) plus a third category (e.g., tetrabenazine, topiramate, or others), before considering DBS.

## The Treatment of ADHD in Children with Tics

ADHD is the most common comorbid disorder seen in children and youth with tics. Rates of comorbidity range from 38% in community‐based studies^64^ to 60% in referral centers.^65^ Given the high pretest probability of an ADHD diagnosis in children who seek medical attention for tics, all children should be screened for this diagnosis. This can easily be accomplished through the use of various parent and teacher‐completed standardized rating scales,^15^ many of which are publically available without a user fee. Making an ADHD diagnosis in children with tics is essential because of the known impact this comorbid disorder has on overall psychosocial functioning.^66^ The risk of aggressive and delinquent behavior in children with tic disorders is largely due to the presence of ADHD and is 1 of the most disabling aspects of the disorder for families.^67^

Children with ADHD exhibit a persistent pattern of inattention and/or hyperactivity that interferes with functioning or development. According to the DSM‐5 criteria for ADHD combined presentation, children must have ≥6 symptoms of inattention and ≥6 symptoms of hyperactivity‐impulsivity that have been present for at least 6 months and are inappropriate for their developmental level.^2^ Children can be diagnosed with a predominantly inattentive presentation if they have enough symptoms of inattention, but not hyperactivity, or with a predominantly hyperactive‐impulsive presentation if the converse is true. Symptoms must be present before age 12 years and must occur in 2 or more settings (i.e., home and school).^2^

The association between tic disorders and ADHD is compelling, and several investigators have proposed that the disorders share a common pathophysiology.^68^ Specifically, both conditions are thought to involve alterations in noradrenergic and dopaminergic transmission, resulting in inadequate modulation of corticostriatal circuits and thus failure to inhibit intrusive thoughts, sensory input, and motor output. If a diagnosis of ADHD is confirmed in a child with tics, then the treatment of ADHD symptoms should be discussed with the child and parents based on the level of associated dysfunction. Serious consideration should be made in children who are failing to perform academically and whose symptoms seriously impair social function or are associated with severe oppositional or aggressive behavior.

The most effective treatments for ADHD and related oppositional and aggressive behaviors are psychostimulants. Psychostimulants block the reuptake of dopamine and norepinephrine into the presynaptic neuron (methylphenidate) or increase the release of these monoamines into the extraneuronal space (amphetamines).^69^ For decades, clinicians were reluctant to use stimulants to treat symptoms of ADHD in children with tics for fear of worsening the tics. In the 1970s and early 1980s, several case reports and small case series were published of children who experienced the onset or worsening of tics after the initiation of stimulants for the treatment of ADHD.^70^, ^71^ Despite new evidence supporting the use of psychostimulants in children with tics and ADHD,^72^ product monographs for stimulants approved for the treatment of ADHD by Health Canada and the US Food and Drug Administration continue to include warnings against the use of these medications in children who have comorbid tic disorders or a family history of TS.

Several trials on the use of psychostimulants and other medications for ADHD symptoms in children with tics have been conducted.^72^ One of the most important trials was conducted by the Tourette Syndrome Study Group,^73^ which randomized 136 children to receive a flexible dose of methylphenidate, clonidine, clonidine plus methylphenidate, or placebo for 16 weeks each. The primary outcome was the change from baseline to Week 16 on the ADHD Conner's Abbreviated Symptoms Questionnaire for Teachers (ASQ); the main secondary outcome was the change from baseline on the YGTSS. A statistically significant treatment effect compared with placebo was observed with methylphenidate alone and with clonidine plus methylphenidate on the ASQ. YGTSS scores also significantly improved compared with placebo, with a statistically significant treatment effect observed for methylphenidate alone and for methylphenidate plus clonidine. In participants who received methylphenidate (either alone or with clonidine), worsening of tics occurred in 20%, which was no more frequent than that observed in participants who received placebo (22%). However, tics limited dosage increases more often in participants who were assigned to methylphenidate alone (35%) than in those who were assigned to methylphenidate plus clonidine (15%), clonidine alone (18%), or placebo (19%). Subsequent trials of methylphenidate have also demonstrated improvements in both ADHD and tic symptoms^74^ in children with both disorders. There has been only 1 small trial of an amphetamine in children with comorbid tics and ADHD. That trial found improvements in ADHD symptoms but worsening of tics when doses higher than 25 mg per day were used.^75^

Psychostimulants should be used as first‐line treatment for ADHD symptoms in children with tics, because the effect sizes associated with their use for ADHD symptoms are much larger than those associated with nonstimulant ADHD treatments. Methylphenidate use should be favored over amphetamines, because there are more data supporting its use in children with comorbid tics. A 20% risk of tic worsening should be discussed with the child and family. Amphetamines can also be tried, with care given to keeping the dosages in the lower range to minimize the chance of tic worsening. Long‐acting stimulant preparations are favored because of their ease of use (once daily dosing) and lower risk of abuse.

If tics are a major concern in the child with comorbid ADHD, then consideration can be given to using first‐line clonidine or guanfacine rather than psychostimulants, because the α2A‐agonists reliably target symptoms of both disorders^72^ as well as related oppositional behavior.^76^ In contrast to the psychostimulants, the α2A‐agonists can take several weeks before noticeable effects on symptoms are apparent, and the amount of improvement in ADHD symptoms is typically less than what is observed with psychostimulants. The α2‐adrenergic agonists can be safely combined with psychostimulant medications, which may allow optimal treatment of both tic and ADHD symptoms.

Oppositional and aggressive behavior related to the diagnosis of ADHD usually responds to pharmacotherapy with ADHD medications, including psychostimulants, α2A‐agonists, and atomoxetine.^76^ If these symptoms persist despite ADHD treatment, then psychosocial interventions, including parent‐training, family therapy addressing parent‐child relationships and communication, and cognitive behavioral therapy (CBT),^77^ are safe and effective treatment strategies and are favored over the use of antipsychotic medications or mood stabilizers.^76^

Before prescribing a psychostimulant, physicians should inquire about a family history of sudden cardiac death or arrhythmias, perform blood pressure and heart rate measurements, and conduct a cardiac examination. Abnormalities should prompt an electrocardiogram and, depending on the outcome, further consultation before initiating the stimulant.

## The Treatment of OCD in Children with Tics

Obsessive‐compulsive behaviors are common in individuals with tics. A comorbid diagnosis of OCD is made in 11% to 66% of individuals with tics, depending on the population studied.^7^, ^65^, ^78^, ^79^, ^80^, ^81^ Clinically, it is often challenging to distinguish compulsions from complex tics, because they can appear similar, and certain behaviors may have aspects of both.^82^, ^83^ In general, however, compulsions are more elaborate and often serve to relieve anxiety associated with an obsession, whereas most tics tend to be performed in response to a feeling of physical tension or “premonitory urge.”^82^, ^83^

The DSM‐5 criteria for OCD specifies the presence of obsessions, compulsions, or both that are time consuming (take more than 1 hour per day) or cause clinically significant distress or impairment in functioning.^2^ Obsessions are defined by recurrent and persistent thoughts, urges, or impulses that are intrusive and unwanted and that cause marked anxiety or distress. Individuals attempt to ignore or suppress obsessions or to neutralize them by performing a compulsion. Compulsions are defined as repetitive behaviors or mental acts that an individual feels driven to perform in response to an obsession or according to rigidly applied rules. The behaviors or mental acts are aimed at preventing or reducing anxiety or at preventing some dreaded event or situation, although they are not connected in a realistic way with what they are designed to neutralize or prevent, or they are clearly excessive. The DSM‐5 includes a new diagnostic subtype for tic‐related OCD based on whether the individual has a current or past history of a tic disorder.^2^

Tic‐related OCD may differ from pure OCD, although there are inconsistencies reported across studies. Tic‐related OCD has an earlier age of onset and is more common in males (as are tic disorders), whereas OCD without tics is more likely to present later and is associated with an equal sex distribution or even a female predominance.^84^, ^85^ Comorbidity patterns also appear to differ, because studies indicate that patients with tic‐related OCD have higher rates of ADHD, other disruptive behavior disorders, trichotillomania, and body dysmorphic disorder.^86^, ^87^ Finally, some evidence suggests that the 2 groups of patients tend to have different types of OCD symptoms.^8^, ^82^, ^83^, ^84^, ^85^, ^88^ Patients with tics appear to have more aggressive, sexual, religious, and symmetry‐related obsessions as well as counting, ordering, touching, blinking, hoarding, and self‐damaging compulsions. Patients without tics, on the other hand, appear to have more obsessive‐compulsive symptoms related to dirt, contamination, and cleaning.

Given the high rate of obsessive‐compulsive behaviors in children with tics, evaluation for the presence of these symptoms should be performed in every child with tics on initial referral and periodically on re‐assessment. Natural history studies suggest that OCD symptoms peak later than tics.^5^ The Children's Yale‐Brown Obsessive Compulsive Scale^16^ can facilitate history taking in this area: The child completes the obsessions and compulsions checklists before the clinic visit, and the clinician reviews the checklist and administers the severity ratings during the clinical encounter.

Current evidence suggests that first‐line treatment of OCD in individuals with tics should be CBT, which is similar to what is recommended for children with OCD only. There is evidence to suggest that individuals with tics may not respond as well those without tics to selective serotonin reuptake inhibitors (SSRIs) for OCD symptoms. This evidence comes from RCTs in which subanalyses were conducted among individuals with tics and from trials that specifically studied youths who had both disorders.^89^, ^90^, ^91^, ^92^

The Pediatric OCD Treatment Study (POTS) was an RCT of 112 youths (ages 7–17 years) with OCD who were randomized to receive treatment with sertraline, OCD‐specific CBT, combined sertraline plus CBT, or placebo. All 3 active treatments were identified as superior to placebo, and the combined treatment was superior to CBT and sertraline alone, which did not differ from one another.^90^ A subanalysis was performed of data from the 17 youths who had comorbid tic disorders (15% of the total sample) in that study. At baseline, the mean OCD symptom severity was not different between individuals with and without a tic disorder. The subanalysis indicated that, in children with tic‐related OCD, sertraline did not differ from placebo for the treatment of OCD symptoms, whereas combined treatment remained superior to CBT alone, and CBT alone remained superior to placebo.^93^

The POTS II trial was an RCT of 124 youths between ages 7 and 17 years with OCD examining the efficacy of CBT augmentation strategies in those who had a partial response to optimal SSRI treatment. Individuals were randomized to receive medication management only, medication management plus CBT augmentation, or medication management plus instruction in CBT skills. The trial demonstrated that those who received medication management plus CBT augmentation had significantly greater symptom reduction compared with those who received medical management alone or medication management plus instruction in CBT skills. In the POTS II trial, 66 of 124 youths in the study had tics (53% of the sample), suggesting that a partial response to optimal SSRI treatment may be more common in this subgroup. OCD symptom severity at baseline did not differ between those with and without tics. Individuals with tic‐related OCD in the study benefitted from CBT to the same degree as those with OCD alone, supporting the use of CBT in this patient population.

Data are scarce on the use of antipsychotic augmentation for OCD symptoms in children, with or without comorbid tic disorders. There is limited evidence from RCTs to support the use of risperidone and aripiprazole as adjunctive treatment to antidepressants in adults with treatment‐resistant OCD.^94^, ^95^, ^96^ The American Psychiatric Association OCD guideline suggests that adults with tic‐related OCD may be more likely to benefit from antipsychotic augmentation than those without tics.^97^ In children with tic‐related OCD, evidence on the use of antipsychotic augmentation comes mainly from case series of children who received treatment with risperidone and aripiprazole. The largest case series of 120 consecutive patients studied the efficacy of augmentation of SSRIs with risperidone or aripiprazole in youths with tic‐related OCD who were nonresponders to SSRI monotherapy.^98^ Children received trials of sertraline or fluvoxamine for at least 12 weeks before randomly receiving augmentation with either risperidone or aripiprazole. Of the 120 children who received an SSRI, 69 did not have an adequate response and were started on risperidone or aripiprazole. The response to antipsychotic augmentation did not differ between agents. Thirty‐nine of the 69 children had clinically important improvements in their OCD symptoms with antipsychotic augmentation. Improvements in tics were observed in 47 of the 69 children.

Although there is not high‐level evidence to support the use of antipsychotic augmentation for OCD symptoms in youths with tic‐related OCD, its use in patients with both disorders makes sense clinically, given the overlapping phenomenology of these disorders and their established efficacy for the treatment of tics. Understanding the known harms of antipsychotic medications, treatment for tic‐related OCD should begin with CBT, and children who do not exhibit an adequate improvement in symptoms can go on to receive pharmacotherapy with an SSRI. OCD symptoms can take at least 12 weeks to improve, and doses at the higher end of the recommended range may be needed. Children who have inadequate improvement on an SSRI or who have severe tics may need the addition of an antipsychotic medication, with risperidone or aripiprazole being the preferred agents. Children should receive appropriate monitoring for both metabolic and extrapyramidal side effects as well as electrocardiograms to measure the QT_c_ interval which can be prolonged by both antipsychotic and antidepressant medications.

## Conclusion

TS is a complex neurodevelopmental disorder, and tics frequently co‐occur with other neuropsychiatric symptoms. Although symptoms may be overwhelming in early life, children and parents should be reassured that many children will have a substantial improvement or even resolution of tics with continued brain development and maturation, and that several symptomatic treatment options are available. The clinician treating the child and family with TS plus must have a holistic approach to assessment and management, because failure to attend to the relevant different aspects of this multifaceted disorder will result in dissatisfaction with care and inadequate treatment. The use of standardized patient‐completed and/or parent‐completed rating scales can assist the busy clinician in collecting relevant information on various symptom domains in a timely fashion. Multidisciplinary teams with expertise in neurology, psychiatry, psychology, and pediatrics may be helpful to ensure that the complex needs of these children are addressed.

## Author Roles

1. Research Project: A. Conception, B. Organization, C. Execution; 2. Statistical Analysis: A. Design, B. Execution, C. Review and Critique; 3. Manuscript Preparation: A. Writing the First Draft, B. Review and Critique.

C.G.: 3A, 3B

D.M.: 3A, 3B

T.P.: 3A, 3B

## Disclosures

**Ethical Compliance Statement:** We confirm that we have read the Journal's position on issues involved in ethical publication and affirm that this work is consistent with those guidelines.

**Funding Sources and Conflicts of Interest:** All authors report no conflict of interest.

**Financial Disclosures for the previous 12 months:** Christos Ganos reports academic research support from the German Research Foundation (GA2031/1‐2) and has received support in the form of a travel grant from the Guarantors of Brain. Davide Martino reports a grants from the European Union (FP7 Programme) and the National Institutes of Health Research (NIHR) of the National Health Service (NHS) of the UK (Research for Patient Benefit programme); he also reports lecturing honoraria and travel support from Merz Pharmaceuticals and from the Movement Disorders Society; and he receives royalties from Oxford University Press and Springer Verlag. Tamara Pringsheim reports research grants from the Canadian Institutes of Health Research, the Mathison Centre for Mental Health Research and Education, the Alberta Mental Health Strategic Clinical Network, the Sick Kids Foundation, and Shire Canada.
